# Identification of the CIPK-CBL family gene and functional characterization of *CqCIPK14* gene under drought stress in quinoa

**DOI:** 10.1186/s12864-022-08683-6

**Published:** 2022-06-16

**Authors:** Zhu Xiaolin, Wang Baoqiang, Wang Xian, Wei Xiaohong

**Affiliations:** 1 College of Agronomy, Gansu Agricultural University, Lanzhou, 730070 China; 2 College of Life Science and Technology, Gansu Agricultural University, Lanzhou, 730070 China; 3 Gansu Provincial Key Laboratory of Aridland Crop Science, Gansu Agricultural University, Lanzhou, 730070 China

**Keywords:** CBL-CIPK, Genome-wide analysis, Drought stress, Gene expression, Quinoa

## Abstract

**Background:**

Calcineurin-like Protein (CBL) and CBL interacting protein kinase (CIPK) play a key role in plant signal transduction and response to various environmental stimuli. Quinoa, as an important plant with high nutritional value, can meet the basic nutritional needs of human Cash crop, is also susceptible to abiotic stress. However, CBL-CIPK in quinoa have not been reported.

**Results:**

In this study, 16 CBL and 41 CIPK genes were identified in quinoa. CBL-CIPK gene shows different intron-exon gene structure and motif, they participate in different biological processes, and form a complex regulatory network between CBL-CIPK proteins. Many cis-regulatory element associated with ABA and drought have been found. The expression patterns of CBL-CIPK showed different expression patterns in various abiotic stresses and tissues. RT-qPCR showed that most members of these two gene families were involved in drought regulation of quinoa, in particular, the expression levels of *CqCIPK11*, *CqCIPK15*, *CqCIPK37* and *CqCBL13* increased significantly under drought stress.

**Conclusions:**

The structures and functions of the CBL-CIPK family in quinoa were systematically explored. Many CBL-CIPK may play vital roles in the regulation of organ development, growth, and responses to abiotic stresses. This research has great significance for the functional characterisation of the quinoa CBL-CIPK family and our understanding of the CBL-CIPK family in higher plants.

**Supplementary Information:**

The online version contains supplementary material available at 10.1186/s12864-022-08683-6.

## Introduction

Under various abiotic stresses (such as drought, low temperature and high salt stress), plants will evolve a set of protective mechanisms to cope with the abiotic stresses and ensure the normal growth and development of plants [1]. Calcium, as the second messenger, plays an important role in plant stress signal transduction. It is widely involved in plant growth and development and regulates the response to environmental stress. Calcium can transform external signals into cytoplasmic information, to further drive a response to a particular stimulus [2]. There are four major calcium ion sensors in plants, including calmodulin (CAMs), calmodulin-like proteins (CMLs), calmodulin-dependent protein kinases (CDPKs) and calcineurin B-like proteins (CBLs), They perceive and modulate the response to calcium concentration of various stimuli [3]. Calcineurin B-like proteins (CBLs), as a unique calcium receptor in plants, can sense the changes of Ca^2+^ in plants and combine with the CBL-interacting protein kinases (CIPKs) to form the CBL-CIPK complex, when stress occurs, the complex is phosphorylated with the corresponding target protein, and the signal is transmitted downstream to improve the stress response ability of the plant under stress [4].

As far as the CBL-CIPK protein complex is concerned, the CBL protein contains four typical helix-loop-helix motifs (EF-hands) for Ca^2+^ binding, the EF-hand is a helix-loop-helix motif containing 12 + X• + Y• + Z•-Y•-X••-Z residues, where the letters represent the ligands involved in metal coordination and dots represent the intervening residues [5]. CIPKs is a family of small plant-specific serine/threonine (Ser/Thr) protein kinase, also known as SnRK3 protein family [6]. CIPKs usually contain a conserved N-terminal kinase domain and C-terminal regulatory domain, and the C-terminal regulatory domain contains a highly conserved NAF/FISL domain, which contains 24 amino acids consisting of highly conserved N, A and F (NAF) or F, I, S and L (FISL), its role is to interact with CBL and release itself from self-inhibition, thereby enabling the substrate to bind to the kinase domain, NAF/FISL is followed by a PPI motif, PPI motif contains about 37 amino acids, which can be combined with PP2C (protein phosphatase), an inhibitor of CIPK kinase activity [7]. The conserved N-terminus is a serine/threonine protein kinase catalytic domain. The N-terminal kinase domain has an ATP binding site and an activation segment. The activation segment contains a phosphorylated activation loop. Amino acid phosphorylation activates the kinase, and the N-terminus of CIPK is similar to the N-terminal kinase domain found in other plant protein kinases, containing a catalytic structure and an activation structure [8]. As target proteins of CBL, CIPKs together with CBLs form a Ca^2+^-mediated CBL-CIPK regulatory network.

Currently, the CIPK-CBL gene has been identified in many species, including Arabidopsis [9], rice [10], apple [11], grape [12], cotton [13], honeysuckle [14], pepper [15], rape [1], tomato [16] and alfalfa [17]. In addition, studies have shown that the regulatory network composed of CIPK-CBL is widely involved in plant stress response and growth and development [18–28]. For example, AtCBL9-AtCIPK3 and AtCBL1-AtCIPK7 signaling pathways are involved in low temperature-induced regulation [18, 19]; AtCBL1/9-AtCIPK23-AKT1 signaling pathway uptake of potassium ions leads to the closure of stomata on plant leaves, indirectly improve the drought resistance of plants [23]. Overexpression of apple *MdCIPK6L* gene in tomato can improve plant drought resistance [22]. Down-regulation of *OsCIPK23* gene expression in rice reduces rice resistance to drought stress [29]; *TaCIPK7*, *TaCIPK15*, *TaCIPK24* and *TaCIPK32* in wheat are involved in plant low temperature stress response [21], and the heterologous expression of *TaCIPK2*, *TaCIPK23* and *TaCIPK27* is enhanced drought resistance of transgenic Arabidopsis plants [25]. Heterologous expression of the *BdCIPK31* gene enhanced the low temperature resistance of transgenic tobacco [26]. CcCIPK14-CcCBL1 in pigeon pea positively regulates plant drought tolerance by enhancing flavonoid biosynthesis [22]. In addition, CIPK-CBL also plays an important role in plant growth and development; AtCBL9-AtCIPK3 is involved in the regulation of abscisic acid (ABA) signaling in seed germination [27], and CBL10-CIPK6 is involved in the regulatory pathway of plant immune signaling [28].

Quinoa (*Chenopodium quinoa* Willd.) is an annual dicotyledonous herb, rich in protein, amino acids, minerals and other nutrients, so it enjoys the title of “super grain” [30]. Quinoa has excellent biological characteristics of cold-tolerance, drought-tolerance, salt-tolerance and barren-tolerance, so it is very suitable for planting in arid and semi-arid areas of northwest China. CBLs and CIPKs, as key components of plant perception and transmission of calcium signals, play a crucial role in plant responses to abiotic stresses [9, 10]. However, the function of the CIPK-CBL gene in quinoa is largely unknown. In 2017, the completion of quinoa genome sequence [30] provided an opportunity to systematically study the quinoa CIPK-CBL gene family. Therefore, in this study, we identified 41 CIPK genes and 16 CBL genes in quinoa by bioinformatics, and studied their phylogeny, gene structure, conserved motifs, promoter elements and expression profile. Furthermore, we investigated the expression patterns of CIPK-CBL members in roots and leaves under drought stress. Our findings will provide valuable references for the further utilization of CBL-CIPK genes for the development of drought-tolerant plants in the context of global land loss, and will facilitate the functional characterization of individual CBLs and CIPKs gene responses to stress and developmental signals.

## Result

### Identification of quinoa CIPK-CBL gene family

In the end, we identified 41 CIPK and 16 CBL members in quinoa and named them *CqCIPK01*-*CqCIPK41* and *CqCBL01*-*CqCBL16* (Table S2). The analysis of physical and chemical properties showed that the number of amino acids of CqCIPK protein is between 276 aa (CqCIPK07) and 613 aa (CqCIPK25), and the molecular weight is 30,943.37 Da (CqCIPK07)-68,335.48 Da (CqCIPK25), which is basically synchronized with the changing trend of the number of amino acids. The isoelectric point is between 5.29 (CqCIPK07) and 9.83 (CqCIPK25). In addition, the instability coefficient is between 30.01 (CqCIPK38) and 47.06 (CqCIPK04), most of which (80.49%) belong to stable proteins (proteins with an instability coefficient less than 40). The fat index is 78.56 (CqCIPK25)-100.53 (CqCIPK23). The amino acid number of CqCBL protein was between 135 (CqCBL15) and 381 (CqCBL10), and the instability coefficient was between 32.40 (CIPK07) and 50.75 (CqCIPK14). The hydrophobic coefficients of 57 CqCIPK-CBL proteins were all less than 0, which indicated that CqCIPK-CBL proteins were hydrophilic proteins. Subcellular localization prediction showed that 18 CqCIPK proteins and 2 CqCBL proteins were located in plasma membrane, 8 CqCIPK proteins and 7 CqCBL proteins were located in microbody, and 8 CqCIPK proteins were located in nucleus, 6 CqCIPK proteins and 7 CqCBL proteins were located in cytoplasm (Table S2).

### Phylogenetic tree construction, gene structure and conservative motif analysis

**Fig. 1:**
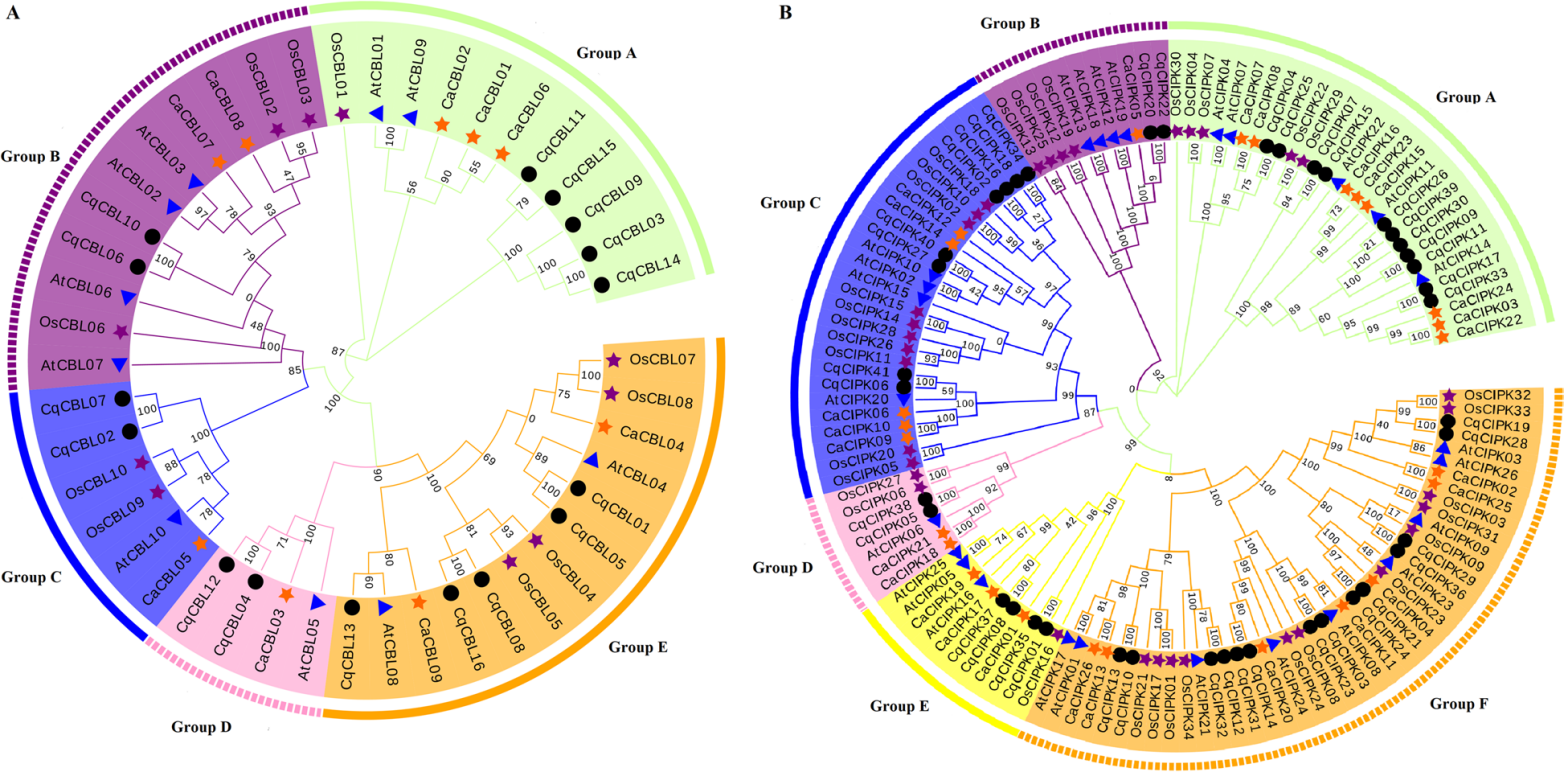
Phylogenetic analysis of CBL (**a**) and CIPK (**b**) protein families in quinoa, capsicum, rice and Arabidopsis. The phylogenetic tree of CBLs and CIPKs was constructed using MEGA-7 based on Neighbor-joining (NJ) method; bootstrap repeats 1000 times. Different subraces are highlighted in different colors. Black solid circles, orange solid pentagons, purple solid pentagons, and blue solid triangles represent CBL and CIPK proteins from quinoa, pepper, rice, and Arabidopsis, respectively

The CIPK-CBL amino acid sequences of quinoa, pepper, rice and *Arabidopsis* (Table S3) were compared and phylogenetic trees were constructed by neighbor-joining method. According to the similarity of sequences, CBL proteins were divided into five subfamilies (Fig. 1A), namely, A, B, C, D and E, each subgroup contains a different percentage of its gene members, and the CIPK proteins of different species were grouped into six subfamilies (Fig. 1B), namely, A, B, C, D, E and F. For the CIPK family, F subfamily has the most CqCIPK members (14), and B and D subfamily have the least CqCIPK members (2). In addition, 20 pairs of paralogous gene were found in quinoa CqCIPK (except *CqCIPK30*), 7 pairs of paralogous gene were found in Arabidopsis AtCIPK and 6 pairs of paralogous gene were found in capsicum CaCIPK. For the CBL family, 7, 2, 2 and 1 pairs of paralogous gene were found in quinoa, Arabidopsis, rice and pepper respectively. There are no orthologous gene pairs between different species, which indicates that the CIPK and CBL genes are highly conserved during the evolution.

**Fig. 2:**
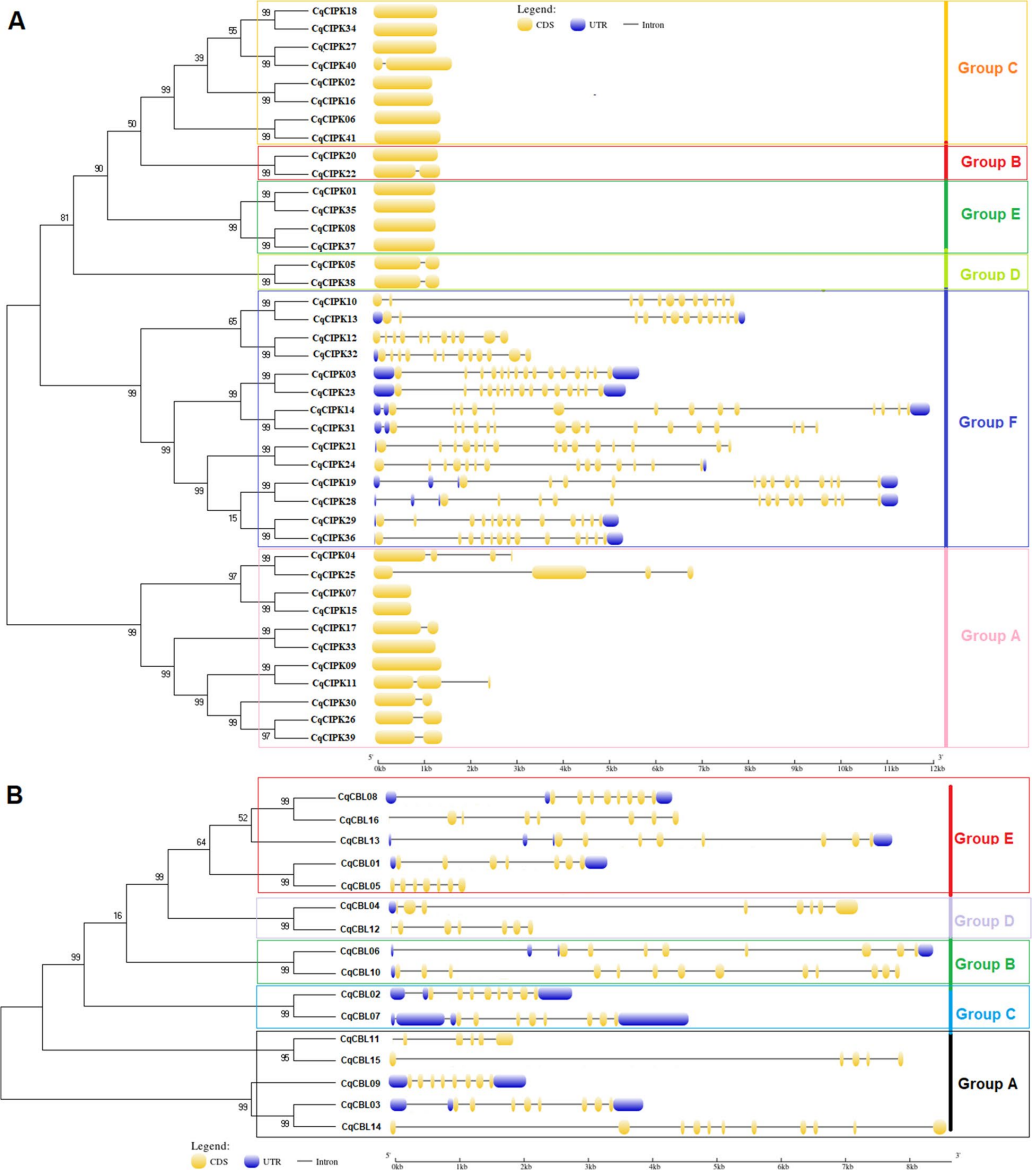
Phylogenetic relationships and gene structure analysis of CBL and CIPK genes in quinoa. The blue boxes represent the 5′-and 3′-untranslated regions; the yellow boxes represent exons; and the black lines represent introns. **A** and **B** represent phylogenetic tree and gene structure of CIPK genes (**A**) and CBL genes (**B**), respectively

The structural analysis of CqCIPK genes showed that the family genes were divided into two types (Fig. 2A): intron-enriched and intron-deleted. The number of exons in A, B, C, D and E is between 1 and 4 and the whole sequence of most genes is exon, such as *CIPK07*, *CIPK09*, *CIPK15* and *CIPK33* in group A, and *CIPK20* in group B, *CIPK02*, *CIPK06*, *CIPK16*, *CIPK18*, *CIPK27*, *CIPK34* and *CIPK41* in group C, *CIPK01*, *CIPK08*, *CIPK35* and *CIPK37* in group E all had only one exon, and all of them had deletion of intron, we think it might have the same function. In contrast, the number of introns in subfamily F ranges from 11 to 15, in addition, most genes have non-coding regions at the 5’and 3’ends. Compared with other subgroups, Subgroup F has more introns and belongs to the intron enrichment class, but the distribution positions on exons are different and the length of sequences are also different. The CqCBL subfamilies also different greatly in gene structure (Fig. 2B). The exons of CqCBL were 5–13, and some genes did not have the non-coding region (*CqCBL05*, *CqCBL11*, *CqCBL12*, *CqCBL14*-*CBL16*).

**Fig. 3:**
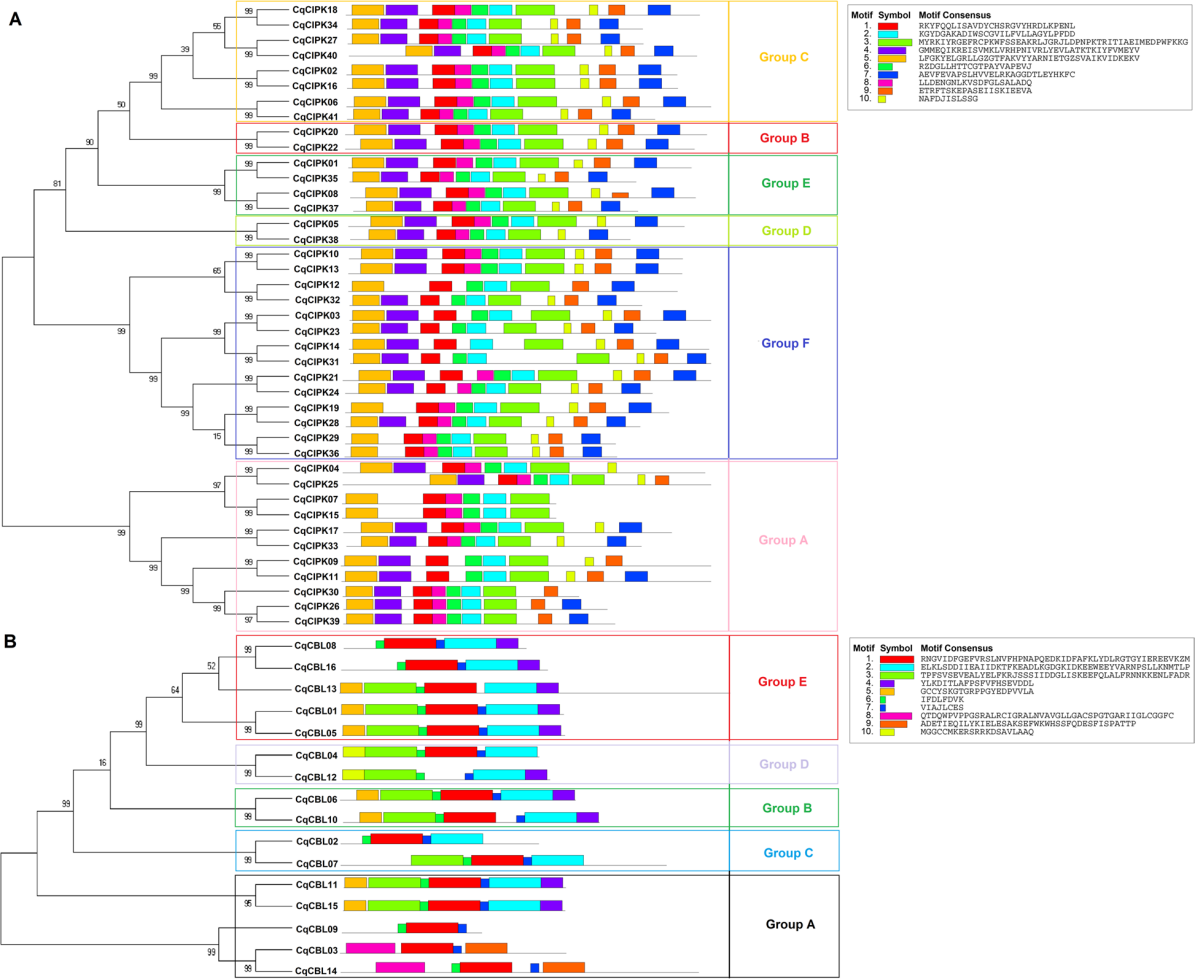
Conservative motifs of CIPK (**a**) and CBL (**b**) in quinoa. The conservative motif sequence is performed using MEME and arranged according to the phylogenetic tree. Different conservative motifs are shown in different color boxes. **A** and **B** represent phylogenetic tree and conserved motifs of CIPK genes (**A**) and CBL genes (**B**), respectively

In addition, we found 10 conservative motifs in the CqCIPK-CBL family. It was found that the motif number of CqCIPK proteins was between 6 and 10 (Fig. 3A). The CqCIPK protein (except CqCIPK41) in group B, C and E all had 10 conservative motifs. 7 CqCIPK proteins in group A had motif numbers between 6 and 9. The motif numbers of 2 CqCIPK proteins in group D were 9. The motif numbers of 14 CqCIPK proteins in group F ranged from 7 to 10. The motif number of CqCBL proteins was 3–7 (Fig. 3B), in which motif 1, motif 6 and motif 7 were found in all CqCBL genes, indicating that these three motifs were highly conserved in 16 CqCBL genes. In addition, we found that the number and location of motifs in different subfamilies are basically the same, and they are highly conservative. We assumed that their domains and functional units are basically similar within the same subfamily.

### Analysis of go enrichment, protein interaction and cis-acting regulatory element

**Fig. 4:**
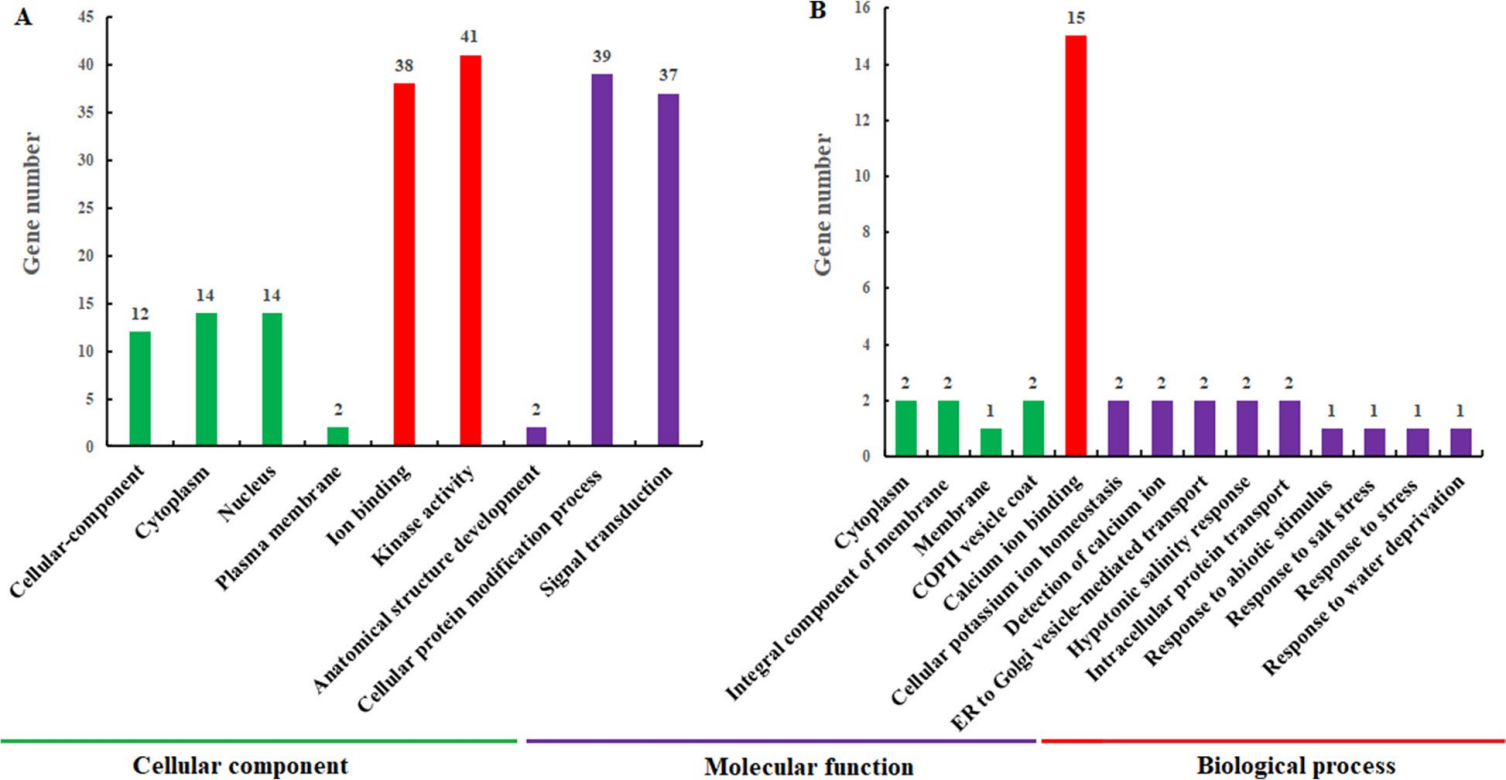
Gene ontology annotation of CqCIPK-CBL proteins in quinoa. **A** Gene ontology annotation of CqCIPK proteins; **B** Gene ontology annotation of CqCBL proteins

The Gene Ontology database was used to analyze the intracellular roles of CqCIPK and CqCBL proteins to comprehensively characterize the properties of CqCIPK and CqCBL genes and gene products. The results showed that CqCIPK-CBL proteins were involved in different functions (Fig. 4A-B), such as molecular function (MF), biological process (BP) and cellular structural component (CC). CqCIPK proteins are mainly involved in cellular-component (GO: 0005575), cytoplasm (GO: 0005737) and nucleus (GO: 0005634), and part of CqCBL proteins are involved in cytoplasm. On molecular function (MF): CqCIPK mainly acts as a protein kinase (GO: 0016301) and binding to ions (GO: 0043167). Interestingly, 41 CIPK proteins have protein kinase activity; 15 CBL proteins have calcium ion binding activity (GO: 0005509). In the aspect of biological process (BP), CqCIPK proteins are mainly involved in anatomical structure development, cellular protein process, signal transduction, and CqCBL proteins are mainly involved in nine biological processes.

**Fig. 5:**
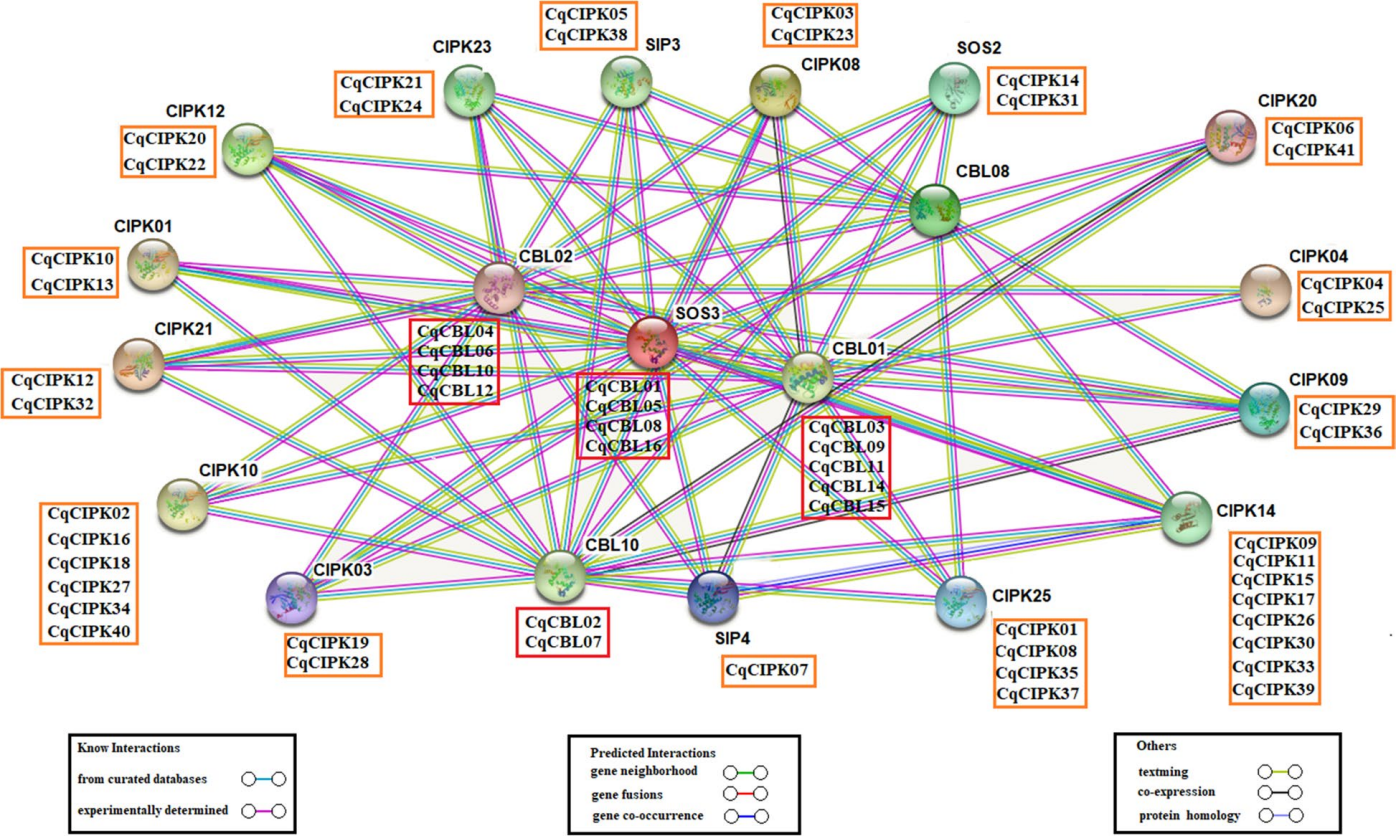
The prediction of the interaction network of CqCBL-CIPK proteins based on the interactions of their orthologs in Arabidopsis

Genes usually carry out their biological functions and signal transduction pathways through interacting networks. Investigation of the potential interaction networks associated with gene families can lead to a better understanding of their function. Studies have shown that CIPK mediates multiple biological processes through its interaction with CBL. Therefore, a possible interaction network of quinoa CIPK-CBL needs to be identified in order to better understand their biological functions. Based on the CIPK-CBL homologous genes of *Arabidopsis thaliana*, the CIPK-CBL protein interaction network of quinoa was constructed by STRING software, and 20 interacting *Arabidopsis thaliana* proteins with high confidence were identified, 41 CIPK proteins and 16 CBL proteins also appear in the known protein-protein network diagram in quinoa (Fig. 5). These proteins that interact with CIPK proteins include calcium-binding CBLS (CBL2, SIP3, SIP4 and CBL10), AKTs associated with potassium transport, and SOSs associated with hydrogen transport. Previous functional analyses have demonstrated that these interactions are involved in plant ion regulation, developmental processes and hormone signal transduction and abiotic stress responses [1, 31].

**Fig. 6:**
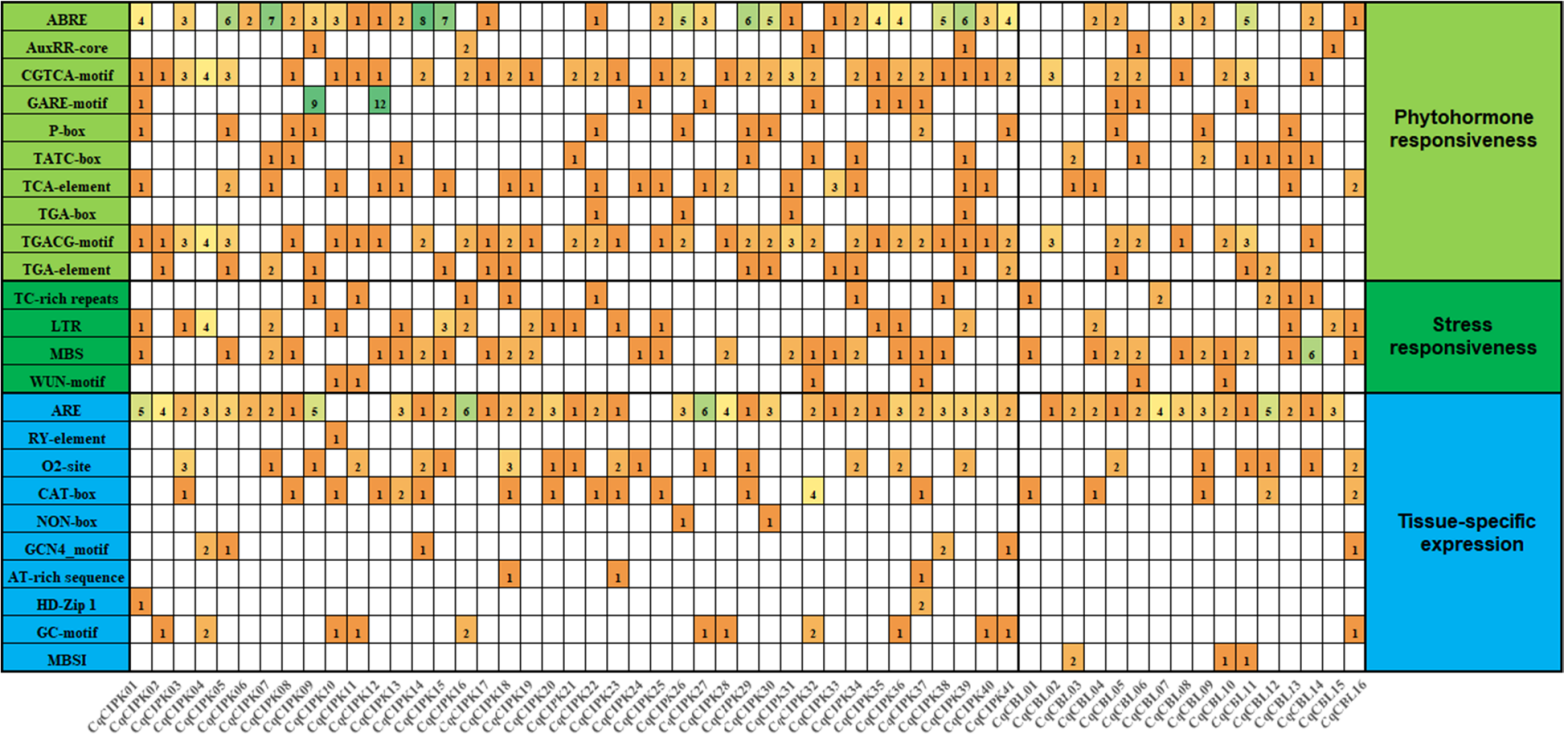
The putative cis-regulatory element and transcription factor binding sites in the promoter region of CBL and CIPK genes of quinoa. The colors and numbers on the grid indicate the number of different cis-regulatory element in the CBL and CIPK genes

Cis-acting regulatory element is a specific motif existing in the promoter region of a gene, which can combine with transcription factors to regulate the downstream genes [32]. Therefore, the identification and analysis of cis-acting regulatory elements in gene promoter regions is helpful to further enhance our understanding of the molecular regulation of these genes. In this study, we analyzed 2000 bp upstream sequences of 41 CqCIPK genes and 16 CBL genes, and found 23 types of cis-acting elements (Fig. 6, Table S4), involving hormone, pressure and tissue-specific expression. Abscisic acid (ABA)-responsive element (ABRE), CGTCA-motif and TGACG-motif (methyl jasmonate response element) were found in the promoter region of most of the CIPK and CqCBL genes. In stress response, TC-rich repeats responded to various defense responses, and existed in single copy in the promoter regions of 7 CIPK genes and 3 CBL genes. LTR (low-temperature responsiveness) element was found in the promoter region of 16 CqCIPK genes and 4 CqCBL genes in response to low temperature. MBS (MYB binding site) element involved drought induction and could be found in the promoter region of 21 CqCIPK genes and 11 CqCBL genes. The promoter region of *CqCBL14* gene contained 6 MBS elements. In tissue-specific expression, ARE (anaerobic induction) element involved in anaerobic induction, existed in the promoter region of 27 CqCIPK genes and 14 CqCBL genes by multiple copies form, CAT-box involved in the expression of meristematic tissue, GCN4-motif involved in the expression of endosperm, MBSI participates in the regulation of flavonoid biosynthesis genes. In addition, RY-element (involved in seed-specific regulation) only exists in *CqCIPK10*, NON-box in *CqCIPK26* and *CqCIPK30*. These results indicated that CqCIPK and CqCBL genes were involved in the growth and stress response of quinoa.

### Expression pattern analysis of CqCIPK-CBL gene in different tissues and treatments

**Fig. 7:**
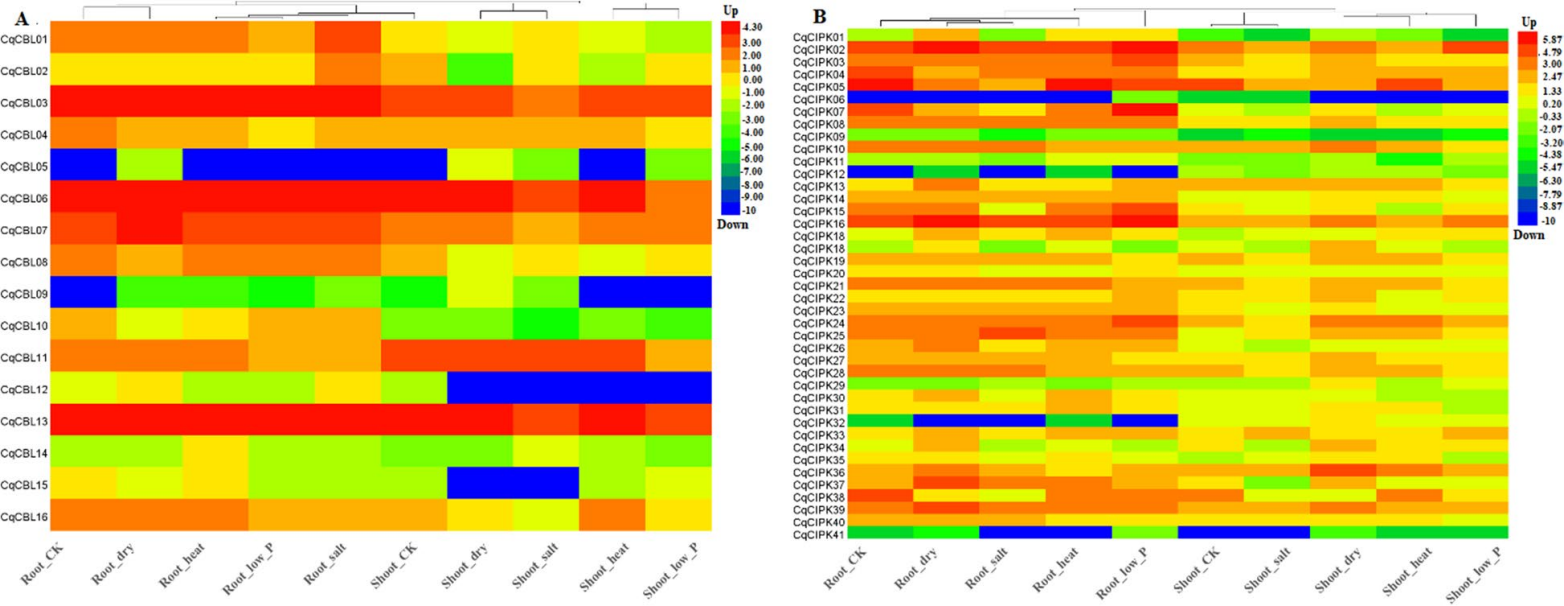
Heatmap of CBL and CIPK genes expression in different treatments. **A** CBL genes; **B** CIPK genes

At the same time, we analyzed the expression patterns of CIPK-CBL family members in roots and shoot apices under different abiotic stresses (Fig. 7A-B, Table S5) using transcriptome data. We found that *CqCBL05*, *CqCBL09*, *CqCIPK06*, *CqCIPK12*, *CqCIPK32* and *CqCIPK41* genes were not expressed in control and in all treatments (salt, drought, high temperature and low phosphorus). In addition, we found that most of the genes (*CqCBL04*, *CqCBL06*, *CqCBL08*, *CqCBL10*, *CqCBL11*, *CqCBL13*, *CqCIPK03*-*CqCIPK05*, *CqCIPK07*, *CqCIPK10*, *CqCIPK14*, *CqCIPK15*, *CqCIPK21*, *CqCIPK24*, *CqCIPK25*, *CqCIPK35*, *CqCIPK36* and *CIPK38*) expressed less in salt, drought and high temperature than control, some genes (*CqCBL02*, *CqCBL03* and *CqCBL07*; *CqCIPK01*, *CqCIPK02*, *CqCIPK08*, *CqCIPK16*, *CqCIPK18* and *CqCIPK37*) were significantly increased under drought stress compared with control, these results suggested that these genes may be involved in drought stress and play a positive regulatory role. The expression of four genes (*CqCBL06*, *CqCBL10*, *CqCIPK05* and *CqCIPK38*) in shoot apex under low phosphorus, drought and salt stress was significantly lower than that in control, drought stress and salt stress play a negative role in regulation. The expression of *CqCIPK02* gene in root and shoot tip was significantly higher than that in control. *CqCIPK02* gene may play a positive role in phosphorus deficiency. The expression of some genes in root and shoot tip (*CqCBL03*, *CqCBL07*, *CqCIPK08*, *CqCIPK16*, *CqCIPK18*, *CqCIPK34*, *CqCIPK36*, *CqCIPK37* and *CqCIPK39*) was significantly higher than that of control under drought stress. These results suggested that CqCIPK-CBL family genes are involved in various abiotic stresses in different ways at the transcriptional level.

**Fig. 8:**
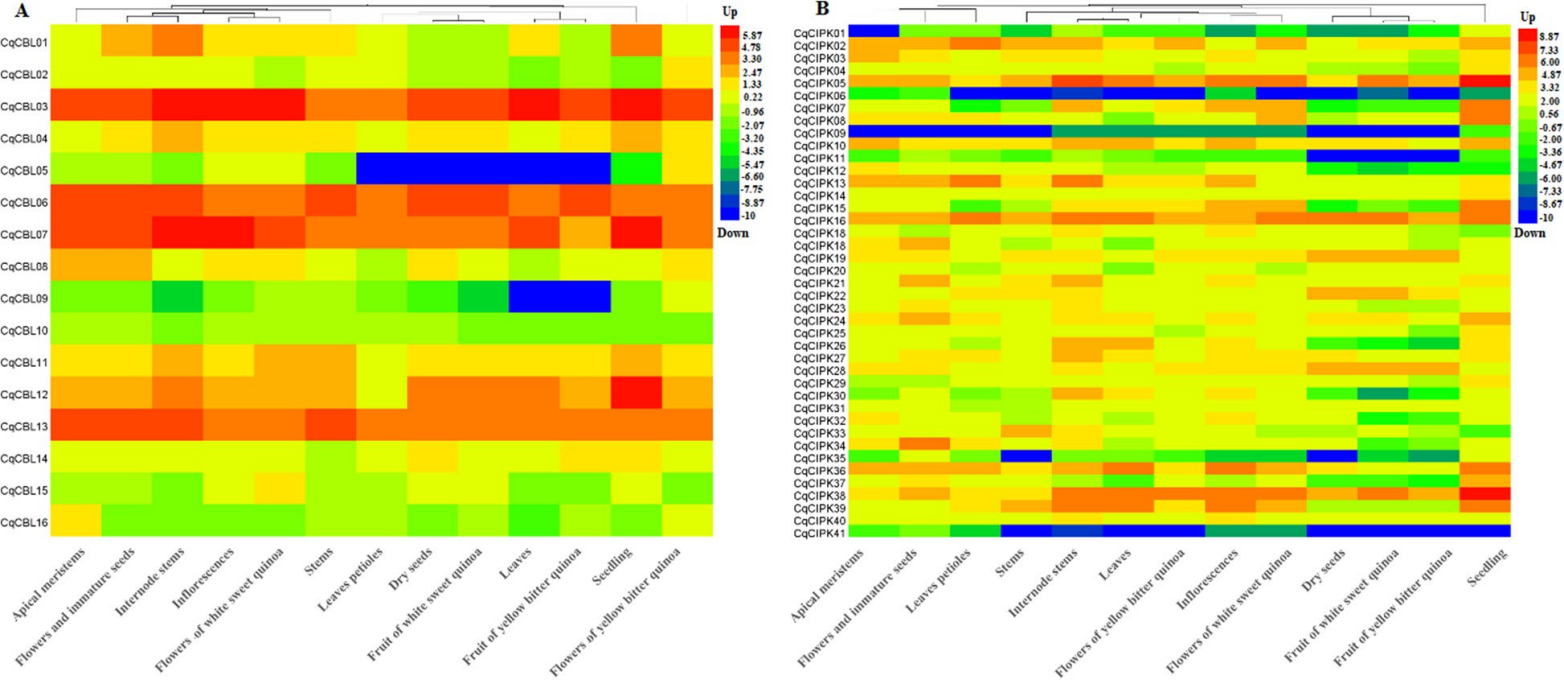
Heatmap of CBL and CIPK genes expression in different tissues. **A** CBL genes; **B** CIPK genes

Studies have shown that CIPK-CBL family genes are involved in different stages of development. Therefore, we studied the expression patterns of the family genes in different tissues and organs by using transcriptome data (Fig. 8A-B). The results showed that most CIPK genes were expressed in all tissues and organs. Among them, *CqCBL05*, *CqCBL09*, *CqCIPK01*, *CqCIPK06*, *CqCIPK09*, *CqCIPK11*, *CqCIPK35* and *CqCIPK41* were almost not expressed in many tissues and organs. *CqCBL03*, *CqCBL13*, *CqCIPK05*, *CqCIPK16* and *CqCIPK38* were highly expressed in all tissues and treatments, especially in internode stems, seedling flowers, flowers of white sweet quinoa, inflows and leaves. *CqCBL15*, *CqCIPK04*, *CqCIPK20* and *CqCIPK31* had relatively low expression abundance in all tissues and treatments. Some genes have tissue specificity (*CqCBL01*, *CqCBL08*, *CqCBL12*, *CqCIPK07*, *CqCIPK12*, *CqCIPK15*, *CqCIPK26* and *CqCIPK30*). For example, CqCBL01 was highly expressed in seedling and almost not expressed in fruit of yellow bitter quinoa, *CqCIPK07* was highly expressed in internode stems, seedling and inflows, but almost not expressed in leaves petioles and dry seeds, and *CqCIPK12* was almost not expressed in fruit of white sweet quinoa, the *CqCIPK15* gene was overexpressed in seedling, inrescuences and flowers of white sweet quinoa, but not expressed in leaves petioles and dry seeds. The *CqCIPK26* gene was overexpressed in internode stems and leaves, but not in fruit of white sweet quinoa and fruit of yellow bitter quinoa. *CqCIPK30* gene was overexpressed in internode stems but not in fruit of white sweet quinoa. In addition, we can observe that the expression patterns of most genes that belong to the lineal homologue are consistent (*CqCBL11*/*CBL15*, *CqCIPK07*/*CIPK15*, *CqCIPK17*/*CIPK33*, *CqCIPK09*/*CIPK11*).

### RT-qPCR validated the expression pattern of CqCIPK-CBL genes in leaves and roots of quinoa under drought stress

**Fig. 9:**
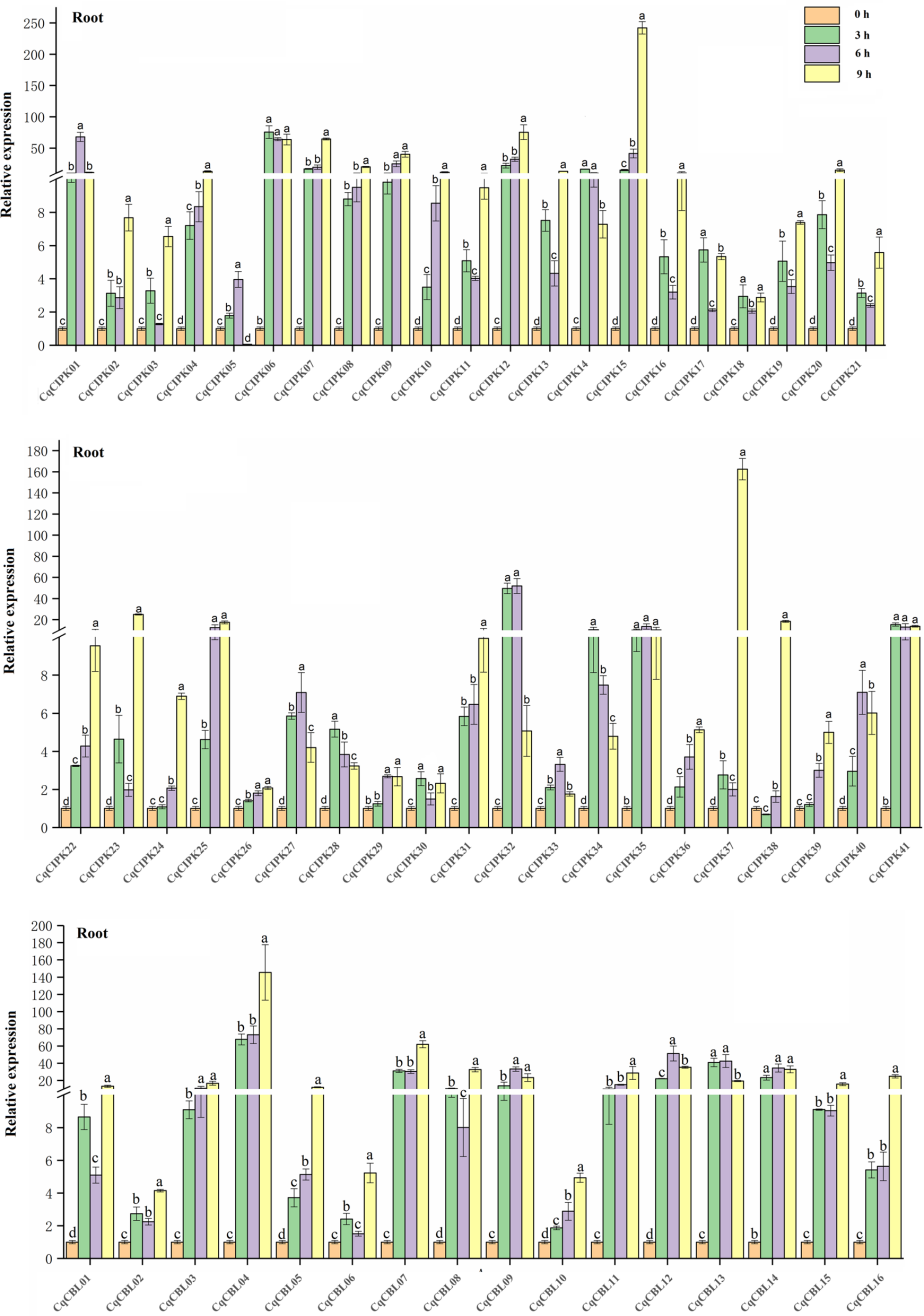
RT-qPCR was used to quantify the expression levels of 16 CBL and 41 CIPK genes from quinoa roots under drought stress. The data is an average of ± SE for three independent biological samples, and the vertical bar represents the standard deviation. All of the expression levels of the CqCIPK-CBL genes were normalized by the expression levels of *CqTUB-9*. Untreated roots (0 h) were normalized as “1” in each graph. Different lowercase letters indicate a significant difference compared to the control (0 h) at *p* < 0.05, respectively

In addition, RT-qPCR was used to detect the expression pattern of CqCIPK-CBL members in quinoa roots under drought stress (Fig. 9). In the roots, all the members responded to drought stress at different time points after drought treatment, and there were significant differences compared with the control, 10 genes (*CqCBL04*, *CqCBL07*, *CqCIPK01*, *CqCIPK06*, *CqCIPK07*, *CqCIPK09*, *CqCIPK12*, *CqCIPK15*, *CqCIPK32* and *CqCIPK37*) were extremely sensitive to drought stress in roots. The relative expression of these 10 genes was at least 40 times that of the control at some point after drought stress, the relative expression of *CqCBL04*, *CqCIPK15* and *CqCIPK37* were 145 times, 242.10 times and 162.53 times, respectively. In the meantime, the relative expression of 25 genes (*CqCBL03*-*CqCBL05*, *CqCBL07*, *CqCBL10*, *CqCBL11*, *CqCBL15*, *CqCBL16*, *CqCIPK04*, *CqCIPK07*-*CqCIPK10*, *CqCIPK12*, *CqCIPK15*, *CqCIPK24*-*CqCIPK26*, *CqCIPK29*, *CqCIPK31*, *CqCIPK36* and *CqCIPK39*) increased with the increase of drought degree. Both reached the maximum value 9 h after drought stress.

**Fig. 10:**
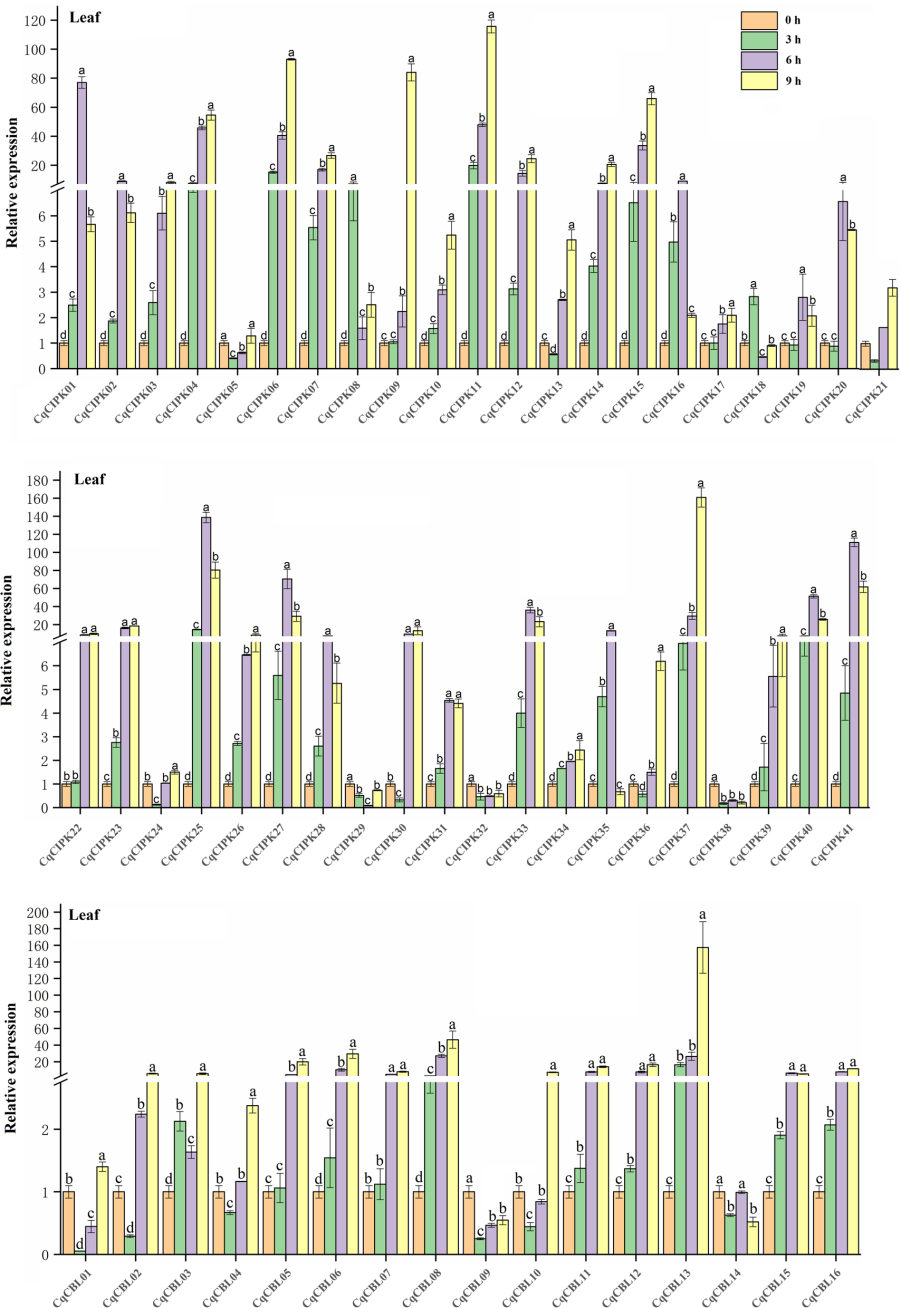
RT-qPCR was used to quantify the expression levels of 16 CBL and 41 CIPK genes from quinoa leaves under drought stress. The data is an average of ± SE for three independent biological samples, and the vertical bar represents the standard deviation. All of the expression levels of the CqCIPK-CBL genes were normalized by the expression levels of *CqTUB-9*. Untreated leaves (0 h) were normalized as “1” in each graph. Different lowercase letters indicate a significant difference compared to the control (0 h) at *p* < 0.05, respectively

In leaves (Fig. 10), the relative expression of a few genes (*CqCBL09*, *CqCBL14*, *CqCIPK05*, *CqCIPK24*, *CqCIPK29*, *CqCIPK32* and *CqCIPK38*) was significantly lower than that of control after drought stress, which indicated that these genes played a negative role in drought stress. At the same time, most of the genes showed two expression patterns under continuous drought stress: The first pattern: the relative expression of 27 genes increased with the increase of drought degree, and reached the maximum 9 h after drought stress. The relative expression of *CqCBL13*, *CqCIPK11* and *CqCIPK37* were 157 times, 115.76 times and 160.79 times, respectively. In the second model, the relative expression of 13 CqCIPK genes increased at first and then decreased with the increase of drought stress, and all of them reached the maximum at 6 h after drought stress. The relative expression of *CqCIPK01* and *CqCIPK25* on the 6 h were 77.06 and 138.53 times, respectively. The relative expression of 6 CqCBL genes were (*CqCBL01*-*CqCBL02*, *CqCBL04, CqCBL09*-*CqCBL10* and *CqCBL14*) decreased first and then increased, and all of them reached the maximum on the 9 h. In addition, we observed an interesting phenomenon, the expression of most CqCIPK genes and CqCBL genes increased sharply in 3–6 h after drought stress (*CqCBL07*, *CqCBL11*, *CqCIPK01*, *CqCIPK02*, *CqCIPK04*, *CqCIPK25*, and *CqCIPK27*), the results indicated that these genes strongly responded to drought stress in the 3–6 h after drought stress.

### Subcellular localization of *CqCIPK14* gene and detection of yeast self-activation

**Fig. 11:**
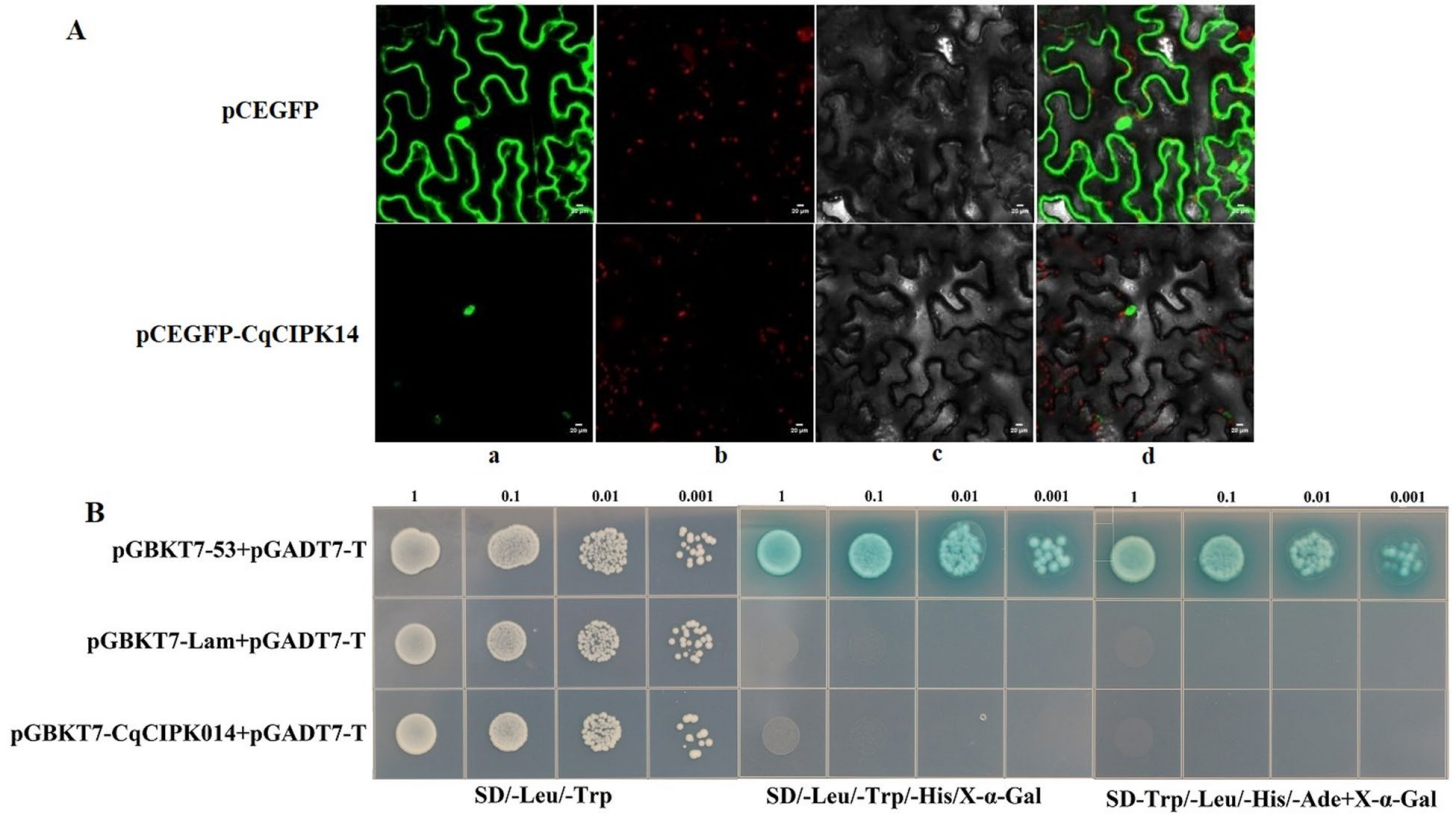
Subcellular localization and yeast self-activation detection. **A** analysis of subcellular localization of CqCIPK14 protein in *Nicotiana tabacum* L. **a** pCEGFP fluorescence signal in the dark field; **b** autofluorescence of chloroplast; **c** cell morphology under bright field; d: combination field. **B** Yeast self-activation test. PGBKT7–53 × PGADT7-T was the positive control, PGBKT7-LAM × PGADT7-T was the negative control and PGBKT7-CIPK014 + PGADT7 was the experimental group.1,0.1,0.01 and 0.001 represent different multiples of dilution of the yeast solution

In order to study the function of *CqCIPK14*, we studied its subcellular localization. *CqCIPK14* was fused with GFP under the 35S promoter and expressed instantaneously in *Nicotiana tabacum L*. The results showed that CqCIPK14-GFP was localized in the nucleus of *Nicotiana tabacum L.* (Fig. 11A). In addition, we performed a transcriptional activation test to study the self-activation activity of *CqCIPK14* (Fig. 11B). PGBKT7–53 × PGADT7-T (positive control), PGBKT7-LAM × PGADT7-T (negative control) and PGBKT7-CIPK14 × PGADT7(experimental group) all grew on SD/−Trp-Leu medium, which indicated that the plasmids were transformed successfully. *CqCIPK014* no grow and turn blue on SD/Trp-Leu-His + x-α-gal medium and SD/Trp-Leu-His-Ade + x-α-gal medium. It is proved that there is no self-activation in CqCIPK14, which can be used in follow-up study.

## Discussion

CIPK-CBL family genes are involved in the regulation of plant responses to biotic and abiotic stresses, and play an important role in many plant life activities [33]. In recent years, the CBL-CIPK pathway in model plants has been studied with great success, but the precise function and mechanism of the CIPK-CBL family genes are still unclear, the study of the CIPK-CBL family genes in quinoa could provide a theoretical basis for further investigation of the interaction between CBL and CIPK proteins in quinoa, and improve the stress resistance of quinoa in molecular breeding and transgenic breeding. Therefore, the whole genome of the CIPK-CBL gene family in quinoa was identified by the bioinformatics method, and 41 CIPK and 16 CBL members were screened by sequence alignment method, which were similar to 43 CIPK genes in maize [34]. The CIPK-CBL gene has been found in many plants: 10 *AtCBL* and 26 *AtCIPK* genes [9] in *Arabidopsis*, there were 10 *OsCBL* and 33 *OsCIPK* genes in rice [10], 8 *VvCBL* and 20 *VvCIPK* genes in grape [12], 9 *CaCBL* and 26 *CaCIPK* genes in pepper [15], 23*MsCBL* and 58 *MsCIPK* genes in *Alfalfa* [17]. Studies have shown that these variations in the number of CBL-CIPK genes between different species are due to events of gene duplication and loss during evolution [35], and that there are multiple CBL-CIPK genes in the quinoa genome, indicating functional diversity of the CBL-CIPK family. In addition, 40 CIPK genes (except *CqCIPK30*) formed 20 pairs of homologous genes, which indicated that most CqCIPK genes were extended in a species-specific manner during the long evolution of plants, this phenomenon has also been extensively demonstrated in the study of other gene families in plants [36].

Intron-exon gene structure is a typical mark of evolution of some gene families [37]. In this study, we found that the gene structure of different subfamily has some similarity. According to the number of introns, 41 CqCIPK genes were classified into two types, intron-enriched type (with nine or more introns) and intron-poor type (without or with only one intron), the intron-deficient gene system occurs after the intron-enriched gene, which may occur when the intron-enriched gene is reinserted into the genome during the reverse transcription process [38]. So far, the same results have been found in maize, poplar, suggesting that the CIPK gene sequence is structurally conserved [10]. The intron loss is faster than the intron gain during gene segment replication, so the intron-enriched CqCIPK may have higher similarity to the original gene. Less intron CqCIPK genes were enriched in group A-E, and more intron genes were clustered in group F, which indicated that the genes of the intron enriched group were closely related to each other, and the genes of the intron deleted group were closely related to each other, this condition is also reported in the cassava *MeCIPK* genes [33], which may be due to gene duplication. These data suggested that intron acquisition and loss events play an important role in the evolution of the CIPK family. It was found that one CqCBL protein could interact with one or more CqCIPK proteins. Similarly, a single CqCIPK protein can interact with one or more CqCBL proteins, consistent with findings in cassava and pepper [33].

Cis-acting regulatory elements, as key molecular switches, are involved in regulating the transcriptional regulation of gene activity in various biological processes [14]. The analysis of promoter region of CqCIPK-CBL genes showed that CqCIPK-CBL genes contained cis-regulatory elements related to environmental stress and hormone induction, which may endow the genes with the potential function of responding to stress and regulating endogenous hormones. When plants are subjected to external stress, such as drought, high temperature, salt and other stress, these stress signals are transformed into signal factors through a series of signals, activating the corresponding transcription factors, and binds to the cis-regulatory element of the corresponding gene, thus activating the expression of the relevant genes in response to external adversity signals [39]. In this study, 10 hormone response elements were found in the promoter region of the CIPK-CBL genes in quinoa. ABRE elements were found in the upstream of 36 CIPK-CBL genes. The ABRE elements were also found in Arabidopsis, rice and honeysuckle [9, 10, 14], it is suggested that CIPK-CBL genes are involved in the ABA signaling pathway, which is mainly involved in stomatal closure, cold, drought and salt stress [14]. In addition, the MBS elements (drought response element) were found in the upstream of 32 CIPK-CBL genes, which was mainly involved in drought-induced response [16]. Therefore, these genes may play a key role in enhancing the resistance of quinoa.

The CIPK-CBL family genes are involved in many stages of plant growth and development [40]. Therefore, the expression patterns of CIPK-CBL family genes in many tissues of quinoa were analyzed by transcriptome data- to better understand their role in plant growth and development. The results showed that 35 CqCIPK and 16 CBL genes were expressed in all the test organs, and the majority of CqCIPK-CBL genes expressed differently in different germplasms of special tissues, which may contribute to the functional diversity of different germplasms of special tissues. In addition, *AtCIPK19* was detected in pollen grains and pollen tubes, especially at much higher levels than in other tissues, and there were differences in tube growth and polarity between *AtCIPK19* overexpression, mutants and complementary lines, indicates that these processes require *AtCIPK19* [41]. In this study, *CqCIPK05*, *CqCIPK16* and *CqCIPK38* were also highly expressed in pollen and inflorescence. The flowering time gene, Gigantea (GI), plays a major role in the control of photoperiod and circadian rhythm. *AtCIPK24* is captured by GI rather than SOS1 under normal conditions, but the complex is degraded in the presence of salt, this provides a unique mechanism between plant development and environmental stress in *Arabidopsis thaliana* [42]. *SlCIPK2* was specifically detected in tomato flower organs and interacted with several SlCBL genes and some stress response transcription factors, indicating that *SlCIPK2* participates in stamen development and stress tolerance through calcium signal transduction [43]. In this study, *CqCBL03*, *CqCBL13*, *CqCIPK02*, *CqCIPK07* and *CqCIPK16* genes were highly expressed in flower organs of different quinoa cultivars. *CqCBL03*, *CqCBL06*, *CqCIPK02*, *CqCIPK16*, *CqCIPK38* and *CqCIPK39* were highly expressed in stem and leaf. This is consistent with CIPK-CBL in *M.domestica* and *O. sativa* [11, 44]. *CqCBL03*, *CqCBL06*, *CqCBL07*, *CqCBL13*, *CqCIPK02*, *CIPK05*, *CIPK10* and *CIPK13* were highly expressed in apical meristems. *CqCBL03*, *CqCBL07*, *CqCIPK02*, *CIPK05* and *CIPK16* were highly expressed in fruits, indicating that they might affect seed size and embryo development, this is consistent with the previous results in *Arabidopsis* and *Prunus mume* [45, 46]. These results suggested that CqCIPK-CBL genes may play an important role in the growth and development of quinoa. In conclusion, the expression profiles of CIPK-CBL genes in different tissues will provide clues for the further study on the development of quinoa.

The CIPK-CBL genes have been shown to play an important role in plant stress, especially in response to drought stress [47]. For example, 4 AtCIPKs (*AtCIPK6*, *− 9*, *− 11*, and *− 23*) and 12 OsCIPKs (*OsCIPK1, − 2, − 5, − 6, − 14, − 17, − 19, − 23, − 24, − 25, − 31,* and *− 32*), *OsCIPK12* and *OsCIPK23* enhance drought tolerance [48]. In *Arabidopsis*, it is found that *AtCIPK16* has a positive regulatory effect on plant salt tolerance, and *AtCIPK23* has a negative regulatory effect on plant drought resistance [49, 50]. *AtCIPK7* regulates cold stress response through interaction with *AtCBL1* [19]. *MtCBL7* was upregulated by PEG, NaCl and ABA stress in *Alfalfa* [48]. Zhao [51] found that *ZmCIPK16* can respond to drought, high salt and ABA induction in a maize study. Piao [52] found that the rice mutant *OsCIPK31* was highly sensitive to drought and high salt stress during seedling growth and seed germination. A small number of genes in this study (*CqCIPK05*, *CqCIPK24*, *CqCIPK29*, *CqCIPK32* and *CqCIPK32*) play a negative regulatory role in leaf drought stress, and similar situations have also occurred in soybeans [53]. Overexpression of *OsCIPK23* in rice can increase the expression level of drought-related genes to improve rice drought tolerance [52]. In this study, the expression of 57 CqCIPK-CBL genes in roots was significantly increased under drought stress, and strongly responded to drought stress, indicating that this family of genes may be involved in drought tolerance in roots, which is consistent with the study in pigeon pea [22]. In addition, previous studies have found that CcCIPK14-CcCBL1 in pigeon pea positively regulates drought tolerance by enhancing the biosynthesis of flavonoids [22], and the absorption of potassium ions by the AtCBL1/9-AtCIPK23-AKT1 signaling pathway in *Arabidopsis* leads to the effects of potassium ions on plant leaves. The stomata are closed, which strengthens the ability of the leaves of the plant to lock water, and indirectly improves the drought resistance of the plant [54]. Down-regulating the expression of *OsCIPK23* gene in rice reduces the resistance of rice to drought stress [29]. The heterologous expression of *TaCIPK2*, *TaCIPK23* and *TaCIPK27* in wheat enhances the drought resistance of transgenic *Arabidopsis* plants through an abscisic acid-dependent pathway [25]. Overexpression of the apple *MdCIPK6L* gene in tomato improves the drought resistance of the plant [55]. In this study, 41 CqCIPK genes directly or indirectly interacted with 16 CqCBL genes, indicating that the responses of these genes to drought stress were also mediated by the CBL-CIPK pathway. The above results suggested that CBL-CIPK can enhance drought tolerance by regulating K^+^ and ABA signaling pathways, which provides a good direction for breeding high-yielding and drought-tolerant crop varieties, it also indicates that there are many drought tolerance regulation mechanisms in different crops.

## Conclusions

In this study, we identified 16 CBL and 41 CIPK genes in quinoa. The functions of these 2 family genes were characterized according to phylogenetic tree, gene structure, conserved sequence, cis-acting regulatory element, GO enrichment and drought stress. It was found that CqCIPK-CBL genes strongly responded to drought stress in roots and leaves. In addition, we further studied subcellular localization and detection of yeast self-activation of *CqCIPK14* gene, and the results showed that CqCIPK14-GFP was localized in the nucleus of *Nicotiana tabacum* L and there is no self-activation in CqCIPK14. Therefore, the results of this study provided a solid foundation for further functional research of CBL-CIPK network.

## Materials and methods

### Identification of quinoa CIPK-CBL family genes

Quinoa genome data and structural annotation information were downloaded from the Phytozome 12 database (https://phytozome.jgi.doe.gov/pz/portal.html). The Arabidopsis, rice, and pepper of CIPK-CBL gene sequences were downloaded from the Plant TAIR database (https://www.arabidopsis.org/) and Ensembl Plants database (http://plants.ensembl.org/). Using Arabidopsis CIPK and CBL gene family members as probes to pre-screen the quinoa CIPK and CBL protein, the E-value value is less than 0.01; Pre-screened biomolecular structure were submitted to the NCBI-swisprot database (http://plants.ensembl.org/) for homology comparison, remove the close-source sequence of quinoa CIPKs, and keep the non-redundant protein sequence. The obtained quinoa CIPK and CBL protein sequence were submitted to Expasy (https://web.expasy.org/compute_pi/) to predict their basic physics and chemical property, such as amino acid numbers, isoelectric point and hydrophobicity. The subcellular location was predicted through WoLF PSORT (http://www.genscript.com/wolf-psort.html).

### Construction of phylogenetic tree, analysis of gene structure and conserved motif of quinoa CIPK-CBL family

Use the MEGA 7 tool to perform sequence alignment on the CIPK and CBL protein sequences of quinoa, Arabidopsis, rice and pepper. Based on the results of multiple sequence alignments, Neighbor-joining (NJ) was used to construct a phylogenetic tree; parameter settings: method was poisson model, the number of bootstrap test was adjusted to 1000, and missing data treatment was adjusted to pairwise deletion.

The GFF annotation information of CqCIPK and CBL genes were submitted to the gene structure display server 2.0(GSDS 2.0, http://gsds.cbi.pku.edu.cn/) for Genetic Structure Analysis. In addition, the amino acid sequences of CqCIPK and CBL proteins were submitted to Multiple Em for Motif Elicitation (MEME, http://meme-suite.org/tools/meme) for analysis of conservative motifs, parameter setting: site distribution is any number of times, conservative the upper limit of the motif is 10, the size of the motif is 10 ~ 60 bp, and other parameters are defaulted.

### GO enrichment, protein interaction network and cis-regulatory element in the promoter region

The CqCIPK-CBL protein sequences were submitted to the blast2go software (https://www.blast2go.com/), and use NCBI-blast as the comparison database to perform GO (gene ontology) functional analysis of the CqCIPK-CBL family genes [56]. Based on the Arabidopsis CIPK homologous genes, software String 10 (http://string-db.org/) was used to predict the protein interaction network map.

The CqCIPK-CBL promoter sequences (2000 bp sequence upstream of the ATG start site) were downloaded from the Phytozome 12 online database (https://phytozome.jgi.doe.gov/pz/portal.html). At the same time, PlantCARE (http://bioinformatics.psb.ugent.be/webtools/plantcare/html/) [57] was used to analyze the types and locations of cis-regulatory element.

### Plant material and treatments

The experiment was conducted in the Plant Physiology Laboratory of the College of Life Science and Technology, Gansu Agricultural University of China from March 2021 to July 2021. The test material was potted seedlings of ‘Long Li No. 2’(from Gansu Academy of Agricultural Sciences). On March 3, 2021, seeds with full grains were selected and sterilized with 5% NaClO for 15 minutes, rinsed with water three times, and thirty seeds were sown in a flower pot with a diameter of 30 cm (Volumen =9.2 L). Mix the turf soil and vermiculite (mixing ratio is 3:1) as a cultivation soil. The culture conditions were: light at 25 °C for 16 h, darkness at 20 °C for 8 h, relative humidity 70–75%, light intensity 450 μmol·m^− 2^·s^− 1^. When the seedlings grow to the 4-leaf stage, robust, consistent and non-polluting seedlings were selected for drought treatment: 20% PEG. The leaves and roots of quinoa seedlings were taken 0 h, 3 h, 6 h and 9 h after treatment. Three biological replicates for each treatment. The leaves and roots were frozen quickly with liquid nitrogen and were placed at − 80 °C for later use.

### Expression pattern analysis, RNA extraction, cDNA synthesis and quantitative PCR

The transcriptome data of different tissues and organs of quinoa (No: PRJNA394651) and different treatments (No: PRJNA306026) were obtained from Bioproject database (www.ncbi.nlm.nih.gov/bioproject). RNA extraction was performed using the plant extraction kit RNAplant-RTR2303 (Zhong ke rui tai Biotechnology Co., Ltd., Beijing) and proceeded according to the operating instructions. Use Agilent 2100 Bioanalyzer and NanoDrop to test the integrity and purity of RNA respectively. After passing the test, RNA was placed at − 80 °C for later use. Primer ScriptTM RT Regent Kit with gDNA Eeaser (TaKaRa, Shanghai) kit was used for reverse transcription to obtain cDNA, and SYBR Primer Ex TaqTM II (TaKaRa) kit was used for quantification. Quantitative PCR instrument is Light Cycler® 96 Real-Time PCR System (Roche, Switzerland). *TUB-9* was taken as an internal reference gene, 57 genes were quantified for RT-qPCR (41 for CIPK and 16CBL), the primer information for 58 genes was listed in Table S1. The reaction system is 20 μL: 1 μL cDNA, 1 μL of the upstream and downstream primers, 10 μL of SYBR, and 7 μL of ddH_2_O. The reaction program is: 95 °C pre-denaturation for 30 s, 95 °C denaturation for 10 s, and 60 °C annealing for 30 s, 72 °C extension 30 s, 40 cycles, repeat 3 times. After the reaction, the fluorescence value change curve and melting curve are analyzed. The relative expression of genes was calculated using 2^-ΔΔCT^ [58].

### Subcellular localization of *CqCIPK14*, transcriptional activation assay of *CqCIPK14*

The coding region of *CqCIPK14* gene was amplified with specific primers. The PCR product was digested with 5′ NcoI and 3′ SpeI. The PCR product was infused and ligated into a pCAMBIA-1302 -EGFP vector under the regulation of the CaMV 35S promoter. After that, the product was transformed into *Agrobacterium tumefaciens* strain GV3101, and empty vector pCAMBIA-1302 -EGFP was used as control (CK). The plasmids were transferred into *Nicotiana tabacum L.* by transient transfection technique [35]. Finally, the spatial expression of CqCIPK-14 protein was observed and recorded by confocal laser scanning microscopy.

*CqCIPK14* was inserted into the pGBKT7 vector using NdeI and BamHI to generate bait construct pGBKT7- CqCIPK14. pGBKT7-Lam and pGBKT7–53 served as negative and positive controls, respectively. Yeast strain AH109 was transformed respectively with the three plasmids and grown in a selection medium lacking tryptophan (SD/−Trp). The positive clones were then obtained and cultured in SD-Trp/−Leu/−His/−Ade + X-α-Gal medium.

## Supplementary Information

**Additional file 1: Table S1.** The primer designed for qRT-PCR.

**Additional file 2: Table S2.** Basic characteristics of CIPK-CBLgenes in quinoa.

**Additional file 3: Table S3.** Amino acid sequences of phylogenetic analysis.

**Additional file 4: Table S4.** Cis-acting elements in the promoter region of CqCIPK-CBL genes.

**Additional file 5: Table S5.** The values of 41 CqCIPK and 16 CqCBL genes in 9 tissues and 5 tretments downloaded from NCBI.

## Data Availability

The reference genome assembly used for data analysis was obtained from National Center for Biotechnology Information (NCBI) BioProject PRJNA675125. The raw transcriptome data generated and analysed in this study deposited in SRA of the NCBI under accession number PRJNA394651 and PRJNA306026. The datasets analysed during this study are included in this published article and its supplementary information files.

## Abbreviations

CDS: Coding sequence length
MEGA: Molecular Evolutionary Genetics Analysis
MEME: Multiple Expectation maximizations for Motif Elicitation
Mw: Protein molecular weight
PEG: Polyethylene glycol
pI: Isoelectric point
RT-qPCR: Real-time quantitative PCR
SMART: Simple Modular Architecture Research Tool
