# Supplementary material for: Identification of the CIPK-CBL family gene and functional characterization of CqCIPK14 gene under drought stress in quinoa
Source: BMC Genomics. 2022 Jun 16;23:447. doi: 10.1186/s12864-022-08683-6 (PMC9204864; doi:10.1186/s12864-022-08683-6)
Supplement: Supplementary file 2 — Additional file 2: Table S2. Basic characteristics of CIPK-CBLgenes in quinoa. [file 12864_2022_8683_MOESM2_ESM.docx]

Table S2 Basic characteristics of CIPK-CBLgenes in quinoa

| Gene accession No | Gene | Size (aa) | Molecular weight (D) | Isoelectric point | Instability index | Aliphatic index | GRAVY | Subcellular Localization |
| --- | --- | --- | --- | --- | --- | --- | --- | --- |
| AUR62012477 | CqCIPK01 | 443 | 50568.06 | 8.92 | 39.88 | 85.96 | -0.254 | plasma membrane |
| AUR62012355 | CqCIPK02 | 427 | 48255.93 | 9.19 | 30.93 | 94.96 | -0.335 | nucleus |
| AUR62019130 | CqCIPK03 | 468 | 53304.36 | 6.10 | 37.42 | 95.56 | -0.178 | plasma membrane |
| AUR62020791 | CqCIPK04 | 468 | 51323.85 | 9.33 | 47.06 | 80.66 | -0.315 | cytoplasm |
| AUR62035950 | CqCIPK05 | 434 | 49007.48 | 7.11 | 30.46 | 86.22 | -0.247 | plasma membrane |
| AUR62027696 | CqCIPK06 | 476 | 54114.02 | 8.04 | 36.65 | 84.75 | -0.420 | plasma membrane |
| AUR62007531 | CqCIPK07 | 276 | 30972.45 | 5.29 | 36.72 | 88.33 | -0.262 | microbody |
| AUR62030835 | CqCIPK08 | 446 | 50699.57 | 8.90 | 35.13 | 80.40 | -0.366 | cytoplasm |
| AUR62031779 | CqCIPK09 | 499 | 57500.20 | 6.35 | 45.14 | 91.80 | -0.435 | plasma membrane |
| AUR62002553 | CqCIPK10 | 431 | 48228.73 | 9.18 | 39.85 | 98.82 | -0.247 | cytoplasm |
| AUR62023980 | CqCIPK11 | 479 | 55355.12 | 8.51 | 42.73 | 96.64 | -0.342 | plasma membrane |
| AUR62040297 | CqCIPK12 | 425 | 48176.57 | 8.96 | 35.13 | 91.72 | -0.310 | nucleus |
| AUR62029970 | CqCIPK13 | 431 | 48391.95 | 9.17 | 39.42 | 98.58 | -0.248 | cytoplasm |
| AUR62013346 | CqCIPK14 | 465 | 53131.26 | 8.79 | 42.12 | 90.32 | -0.204 | nucleus |
| AUR62004023 | CqCIPK15 | 276 | 30943.37 | 5.29 | 38.45 | 86.92 | -0.277 | microbody |
| AUR62003433 | CqCIPK16 | 427 | 48270.00 | 9.20 | 30.65 | 96.11 | -0.312 | nucleus |
| AUR62006359 | CqCIPK17 | 424 | 48494.47 | 8.33 | 33.92 | 82.24 | -0.434 | cytoplasm |
| AUR62006356 | CqCIPK18 | 457 | 51853.11 | 8.79 | 35.01 | 91.05 | -0.307 | nucleus |
| AUR62037145 | CqCIPK19 | 418 | 47396.19 | 5.84 | 38.76 | 82.75 | -0.407 | plasma membrane |
| AUR62012824 | CqCIPK20 | 467 | 53223.29 | 8.62 | 35.99 | 82.61 | -0.271 | plasma membrane |
| AUR62002685 | CqCIPK21 | 512 | 57126.16 | 9.08 | 41.71 | 90.62 | -0.224 | plasma membrane |
| AUR62021832 | CqCIPK22 | 451 | 50613.40 | 8.22 | 41.31 | 85.12 | -0.265 | plasma membrane |
| AUR62014389 | CqCIPK23 | 473 | 53889.29 | 6.34 | 38.74 | 100.53 | -0.154 | plasma membrane |
| AUR62030142 | CqCIPK24 | 475 | 52912.69 | 9.41 | 41.32 | 94.19 | -0.145 | nucleus |
| AUR62043637 | CqCIPK25 | 613 | 68335.48 | 9.83 | 45.95 | 78.56 | -0.400 | nucleus |
| AUR62036220 | CqCIPK26 | 408 | 45861.30 | 8.91 | 32.52 | 94.14 | -0.219 | microbody |
| AUR62036223 | CqCIPK27 | 457 | 51710.39 | 8.03 | 38.82 | 84.70 | -0.435 | plasma membrane |
| AUR62025068 | CqCIPK28 | 455 | 51783.42 | 6.26 | 39.48 | 86.09 | -0.370 | plasma membrane |
| AUR62022663 | CqCIPK29 | 418 | 47457.96 | 8.14 | 37.73 | 79.02 | -0.484 | microbody |
| AUR62044361 | CqCIPK30 | 364 | 41401.13 | 9.11 | 33.44 | 89.75 | -0.217 | microbody |
| AUR62011141 | CqCIPK31 | 569 | 65111.22 | 7.21 | 36.33 | 87.86 | -0.106 | plasma membrane |
| AUR62039991 | CqCIPK32 | 452 | 51152.60 | 7.08 | 36.80 | 87.79 | -0.346 | nucleus |
| AUR62029687 | CqCIPK33 | 456 | 52190.59 | 7.60 | 31.07 | 79.89 | -0.484 | microbody |
| AUR62029685 | CqCIPK34 | 457 | 51642.81 | 8.87 | 37.25 | 89.34 | -0.316 | endoplasmic reticulum |
| AUR62031603 | CqCIPK35 | 443 | 50453.80 | 8.96 | 38.77 | 85.51 | -0.270 | plasma membrane |
| AUR62004550 | CqCIPK36 | 421 | 47554.04 | 6.35 | 36.21 | 81.47 | -0.413 | microbody |
| AUR62026995 | CqCIPK37 | 440 | 50071.71 | 8.75 | 30.80 | 80.84 | -0.364 | cytoplasm |
| AUR62022521 | CqCIPK38 | 433 | 48948.41 | 7.11 | 30.01 | 86.21 | -0.252 | plasma membrane |
| AUR62009727 | CqCIPK39 | 420 | 47488.99 | 8.54 | 31.26 | 90.95 | -0.250 | microbody |
| AUR62009725 | CqCIPK40 | 538 | 60662.50 | 7.05 | 36.36 | 88.09 | -0.331 | plasma membrane |
| AUR62031224 | CqCIPK41 | 476 | 54097.10 | 8.48 | 36.08 | 83.74 | -0.423 | plasma membrane |
| AUR62004495 | CqCBL01 | 198 | 22561.59 | 4.38 | 34.26 | 81.16 | -0.282 | cytoplasm |
| AUR62006092 | CqCBL02 | 214 | 24540.13 | 4.68 | 41.04 | 94.72 | -0.146 | cytoplasm |
| AUR62007176 | CqCBL03 | 213 | 24428.77 | 4.81 | 32.40 | 87.84 | -0.192 | microbody |
| AUR62009157 | CqCBL04 | 313 | 35781.34 | 5.16 | 38.10 | 109.01 | 0.088 | plasma membrane |
| AUR62010856 | CqCBL05 | 189 | 21794.58 | 4.56 | 46.55 | 82.01 | -0.268 | microbody |
| AUR62017129 | CqCBL06 | 256 | 28402.52 | 4.93 | 41.92 | 96.67 | -0.139 | plasma membrane |
| AUR62018829 | CqCBL07 | 213 | 24428.77 | 4.81 | 32.40 | 87.84 | -0.192 | microbody |
| AUR62019400 | CqCBL08 | 214 | 24526.14 | 4.68 | 38.63 | 95.19 | -0.134 | cytoplasm |
| AUR62019854 | CqCBL09 | 199 | 23082.91 | 4.71 | 38.05 | 72.46 | -0.426 | microbody |
| AUR62019855 | CqCBL10 | 381 | 43389.72 | 5.69 | 33.99 | 89.08 | -0.197 | cytoplasm |
| AUR62022068 | CqCBL11 | 190 | 22254.28 | 4.87 | 46.95 | 95.95 | -0.219 | microbody |
| AUR62023458 | CqCBL12 | 175 | 20340.18 | 4.69 | 35.71 | 89.54 | -0.333 | cytoplasm |
| AUR62035680 | CqCBL13 | 226 | 26228.92 | 4.99 | 41.94 | 91.86 | -0.270 | microbody |
| AUR62036054 | CqCBL14 | 344 | 38418.97 | 4.84 | 50.75 | 81.34 | -0.427 | microbody |
| AUR62037340 | CqCBL15 | 135 | 15854.98 | 4.58 | 39.69 | 91.70 | -0.456 | cytoplasm |
| AUR62042203 | CqCBL16 | 217 | 24587.28 | 5.19 | 40.05 | 96.64 | -0.085 | cytoplasm |

| **Gene accession No** | **Gene** | **Alpha helix（Hh）** | **Extended strand（Ee）** | **Random coil（Cc）** |
| --- | --- | --- | --- | --- |
| **AUR62012477** | **CqCIPK01** | **160（36.12%）** | **108（24.38%）** | **175（39.50%）** |
| **AUR62012355** | **CqCIPK02** | **154（36.07%）** | **74（17.33%）** | **199（46.60%）** |
| **AUR62019130** | **CqCIPK03** | **161（34.40%）** | **95（20.30%）** | **212（45.30%）** |
| **AUR62020791** | **CqCIPK04** | **139（29.70%）** | **99（21.15%）** | **230（49.15%）** |
| **AUR62035950** | **CqCIPK05** | **179（41.24%）** | **83（19.12%）** | **172（39.63%）** |
| **AUR62027696** | **CqCIPK06** | **141（29.62%）** | **95（19.96%）** | **240（50.42%）** |
| **AUR62007531** | **CqCIPK07** | **92（33.33%）** | **65（23.55%）** | **119（43.12%）** |
| **AUR62030835** | **CqCIPK08** | **155（34.75%）** | **84（18.83%）** | **207（46.41%）** |
| **AUR62031779** | **CqCIPK09** | **200（40.08%）** | **89（17.84%）** | **210（42.08%）** |
| **AUR62002553** | **CqCIPK10** | **162（37.59%）** | **72（16.71%）** | **197（45.71%）** |
| **AUR62023980** | **CqCIPK11** | **181（37.79%）** | **76（15.87%）** | **222（46.35%）** |
| **AUR62040297** | **CqCIPK12** | **171（40.24%）** | **59（13.88%）** | **195（45.88%）** |
| **AUR62029970** | **CqCIPK13** | **160（37.12%）** | **71（16.47%）** | **200（46.40%）** |
| **AUR62013346** | **CqCIPK14** | **171（36.77%）** | **117（25.16%）** | **177（38.06%）** |
| **AUR62004023** | **CqCIPK15** | **92（33.33%）** | **64（23.19%）** | **120（43.48%）** |
| **AUR62003433** | **CqCIPK16** | **150（35.13%）** | **75（17.56%）** | **202（47.31%）** |
| **AUR62006359** | **CqCIPK17** | **135（31.84%）** | **72（16.98%）** | **217（51.18%）** |
| **AUR62006356** | **CqCIPK18** | **152（33.26%）** | **84（18.38%）** | **221（48.36%）** |
| **AUR62037145** | **CqCIPK19** | **148（35.41%）** | **68（16.27%）** | **202（48.33%）** |
| **AUR62012824** | **CqCIPK20** | **147（31.48%）** | **103（22.06%）** | **217（46.47%）** |
| **AUR62002685** | **CqCIPK21** | **214（41.80%）** | **70（13.67%）** | **228（44.53%）** |
| **AUR62021832** | **CqCIPK22** | **133（29.49%）** | **85（18.85%）** | **233（51.66%）** |
| **AUR62014389** | **CqCIPK23** | **183（38.69%）** | **84（17.76%）** | **206（43.55%）** |
| **AUR62030142** | **CqCIPK24** | **202（42.53%）** | **64（13.47%）** | **209（44.00%）** |
| **AUR62043637** | **CqCIPK25** | **164（26.75%）** | **134（21.86%）** | **315（51.39%）** |
| **AUR62036220** | **CqCIPK26** | **105（25.74%）** | **87（21.32%）** | **216（52.94%）** |
| **AUR62036223** | **CqCIPK27** | **176（38.51%）** | **76（16.13%）** | **205（44.86%）** |
| **AUR62025068** | **CqCIPK28** | **169（37.14%）** | **75（16.48%）** | **211（46.37%）** |
| **AUR62022663** | **CqCIPK29** | **152（36.36%）** | **55（13.16%）** | **211（50.48%）** |
| **AUR62044361** | **CqCIPK30** | **87（23.84%）** | **90（24.66%）** | **188（51.51%）** |
| **AUR62011141** | **CqCIPK31** | **174（30.58%）** | **145（25.48%）** | **250（43.94%）** |
| **AUR62039991** | **CqCIPK32** | **187（41.37%）** | **76（16.81%）** | **189（41.81%）** |
| **AUR62029687** | **CqCIPK33** | **162（35.53%）** | **73（16.01%）** | **221（48.46%）** |
| **AUR62029685** | **CqCIPK34** | **149（32.60%）** | **95（20.79%）** | **213（46.61%）** |
| **AUR62031603** | **CqCIPK35** | **158（35.67%）** | **101（22.80%）** | **184（41.53%）** |
| **AUR62004550** | **CqCIPK36** | **144（34.20%）** | **63（14.96%）** | **214（50.83%）** |
| **AUR62026995** | **CqCIPK37** | **136（30.91%）** | **87（19.77%）** | **217（49.32%）** |
| **AUR62022521** | **CqCIPK38** | **176（40.65%）** | **82（18.94%）** | **175（40.42%）** |
| **AUR62009727** | **CqCIPK39** | **101（24.05%）** | **101（24.05%）** | **218（51.90%）** |
| **AUR62009725** | **CqCIPK40** | **203（37.73%）** | **102（18.96%）** | **233（43.41%）** |
| **AUR62031224** | **CqCIPK41** | **137（28.78%）** | **92（19.33%）** | **247（51.89%）** |
